# Mutations in the NXF-1:NXT-1 mRNA export complex affect gene-expression driven by the *hsp-16.41* promoter

**DOI:** 10.17912/micropub.biology.000918

**Published:** 2023-07-30

**Authors:** Michael W Crawford, Galen Posch, Jerôme Cattin-Ortolá, Irini Topalidou, Michael Ailion

**Affiliations:** 1 Department of Biochemistry, University of Washington, Seattle, WA USA

## Abstract

The NXF-1 : NXT-1 heterodimer is essential for the nuclear export of mRNA. Here we describe three new alleles of *nxf-1* and one allele of *nxt-1* isolated from a forward genetic screen. These mutations cause no apparent phenotype under normal growth conditions, but partially suppress the lethality caused by heat-shock induced expression of the PEEL-1 toxin from *P hsp-16.41 :: peel-1* . There is also decreased expression of *P hsp-16.41 ::eGFP* in an *nxf-1* mutant. We propose that NXF-1 : NXT-1 influences the expression of heat-shock activated genes due to a role in the recruitment of the *hsp-16.41* promoter to the nuclear pore complex during heat-shock.

**Figure 1. Missense mutations in nxf-1 and nxt-1 partially suppress P hsp-16.41 :: peel-1 and P hsp-16.41 ::eGFP:: his-44 but not endogenous peel-1:**
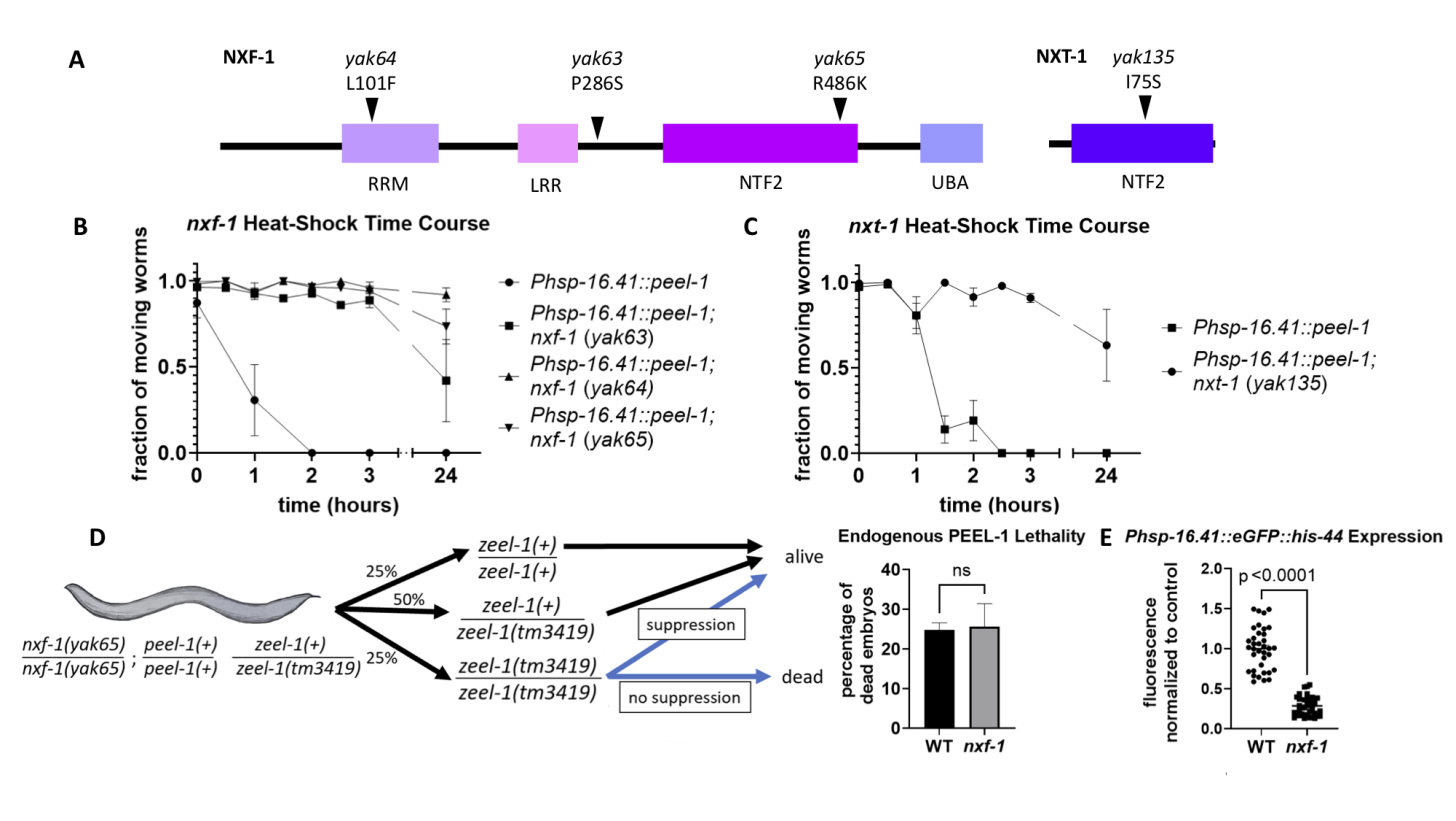
(A) Schematic of NXF-1 and NXT-1 indicating functional domains (see text for details) and the mutations that were identified in our screen. (B-C) Quantification of *P hsp-16.41 :: peel-1* lethality as the fraction of moving (living) worms at different times after a 2 hr heat shock at 34°C; n=3-5, each replicate represents 50 animals; error bars represent standard error of the mean. (D) Schematic of expected outcomes and quantification of dead progeny from animals heterozygous for the *zeel-1 ( tm3419 )* deletion and homozygous for *nxf-1 ( yak65 )* with endogenously expressed *peel-1* . WT control shows quantification of dead progeny from animals heterozygous for *zeel-1 ( tm3419 )* . n=7-9, each n represents 12-127 animals; ns – not significant (p>0.05); error bars represent standard deviation. (E) Relative fluorescence of *P hsp-16.41 ::GFP* in an *nxf-1 ( yak65 )* mutant normalized to control; n=3 replicates, each replicate group represents 8-17 animals; bars represent means. Statistics: unpaired two-tailed t tests.

## Description

PEEL-1 is a toxin found in the sperm of *C. elegans* which is lethal to embryos lacking the antidote ZEEL-1 , and PEEL-1 is also toxic to adult worms when expressed ectopically (Seidel et al., 2008, 2011) . The mechanism of this lethality is not yet known. To better understand how PEEL-1 kills, we performed a genetic screen for suppressors of the lethality caused by *peel-1* expression driven by the heat-shock promoter *hsp-16.41* . Among the mutants found in this screen were four recessive partial suppressors. Three mutants ( *yak63* , *yak64* , and *yak65* ) were mapped to chromosome V, and one to chromosome I *( yak135* ). By whole-genome sequencing we found that *yak63* , *yak64* , and *yak65* are missense mutations in *nxf-1* and that *yak135* is a missense mutation in *nxt-1* ( Figure 1A ).

NXF-1 and NXT-1 are nuclear export receptors which form a heterodimer essential for the nuclear export of mRNA (Katahira, 2015) . NXF-1 contains four domains characterized in human, Drosophila, and yeast orthologs, whose functions are likely conserved in *C. elegans* : an RNA recognition motif (RRM) and a leucine rich repeat (LRR) region which interact with RNA and export adaptors (Katahira, 2015; Katahira et al., 2009; Viphakone et al., 2012) ; a nuclear transport factor 2 (NTF2)-like domain which interacts with the NTF2-like domain of NXT-1 to heterodimerize (Suyama et al., 2000) ; and a ubiquitin-associated (UBA)-like fold which acts with the NTF2-like domain to interact with the phenylalanine-glycine repeats found in nucleoporins (Fribourg et al., 2001) ( Figure 1A ). These domains work in concert to form interactions between NXF-1 : NXT-1 , mRNA, nucleoporins, and components of the TRanscription-EXport complexes TREX and TREX-2 (Fribourg et al., 2001; Katahira et al., 2009; Viphakone et al., 2012; Wickramasinghe et al., 2010) . These interactions link mRNA transcription and processing to nuclear export.

Three of the four alleles from our screen map to known functional domains within the NXF-1 : NXT-1 complex. *nxf-1 ( yak64 )* causes an L101F substitution within the RRM of NXF-1 , *nxf-1 ( yak65 )* causes an R486K substitution within the NTF2-like domain of NXF-1 , and *nxt-1 ( yak135 )* causes an I75S substitution in NXT-1 ’s only functional domain, the NTF2-like domain ( Figure 1A ). *nxf-1 ( yak63 )* maps outside a known functional domain and causes a P286S substitution in the region between the LRR and NTF2-like domains of NXF-1 .

We quantified the partial-suppression of the *nxf-1* and *nxt-1* mutants by performing a heat-shock time-course experiment. Expression of *P hsp-16.41 :: peel-1* was induced by heat shock, and death was measured by response to touch. All wild-type control animals died within the first three hours, while more than 80% of *nxf-1* and *nxt-1* mutant animals remained alive ( Figure 1B,C ). At 24 hours after heat shock, survival ranged from 42-92%. Notably, *nxf-1 ( yak63 )* conferred the least resistance to *P hsp-16.41 :: peel-1* , and this allele is the only one of the four found outside of any known functional domain.

We then asked whether these partial suppressors could also suppress endogenously expressed *peel-1* . *nxf-1 ( yak65 )* was chosen due to its relatively strong suppression of *P hsp-16.41 :: peel-1* lethality. *nxf-1 ( yak65 )* animals heterozygous for a deletion of the PEEL-1 antidote *zeel-1 ( tm3419 )* were generated and allowed to self-reproduce. For controls, we generated *zeel-1 ( tm3419 )/+* animals and selfed them. Mendelian independent assortment predicts that 25% of the progeny would be *zeel-1 ( tm3419 )* homozygotes and would succumb to endogenous PEEL-1 toxicity unless suppressed by *nxf-1 ( yak65 )* . Animals with at least one copy of the antidote *zeel-1 (+)* would remain unaffected by PEEL-1 (Seidel et al., 2008) . We found that about 25% of the progeny of *zeel-1 ( tm3419 )/+; nxf-1 ( yak65 )* animals succumbed to endogenous *peel-1* , matching controls ( Figure 1D ). Thus, although *nxf-1 ( yak65 )* suppresses *peel-1* lethality when the toxin is expressed ectopically under the heat-shock promoter *hsp-16.41* , *nxf-1 ( yak65 )* does not suppress endogenously expressed *peel-1* . We also generated *nxf-1 ( yak65 )* mutants carrying *P exp-3 :: peel-1* which expresses PEEL-1 in the egg-laying muscles (Seidel et al., 2011) . Eight out of nine control *P exp-3 :: peel-1* animals in a wild-type background had an observable egg-laying defect as expected for egg-laying muscle death caused by PEEL-1 . Similarly, seven of nine *nxf-1 ( yak65 ); P exp-3 :: peel-1* animals also had egg-laying defects, indicating that *nxf-1 ( yak65 )* does not suppress PEEL-1 when expressed in egg-laying muscles. These results suggest that the suppression of *P hsp-16.41 :: peel-1* by mutations in *nxf-1* and *nxt-1* is due to an effect on the heat-shock-induced expression of *peel-1* rather than suppression of PEEL-1 toxicity per se.

To confirm that *nxf-1 ( yak65 )* affects the expression of heat-shock induced genes, we generated *nxf-1 ( yak65 )* animals expressing GFP under the *hsp-16.41* promoter. The average fluorescence of these worms upon heat-shock was reduced by 72% compared to worms with wild-type *nxf-1* ( Figure 1E ), further supporting the idea that *nxf-1* affects expression from the *hsp-16.41* promoter.

Because NXF-1 : NXT-1 appears to specifically affect expression of genes driven by the *hsp-16.41* promoter, the NXF-1 : NXT-1 complex appears to be acting at the level of the DNA in contrast to its more widely described role in exporting mRNA. A plausible mechanism is that NXF-1 : NXT-1 may be involved in recruiting the *hsp-16.41* promoter to nuclear pores for efficient transcription and export of mRNA driven by this promoter. In *C. elegans* , localization of the bidirectional heat-shock promoter *hsp-16.2 /41* at the nuclear pore complex was shown to occur during heat-shock (Rohner et al., 2013) . This localization depended on a component of the TREX-2 complex, which was proposed to act as a tether between the *hsp-16.2 /41* promoter and the nuclear pore complex (Rohner et al., 2013) . Interestingly, the human TREX-2 component GANP directly interacts with NXF1 (Wickramasinghe et al., 2010) , suggesting that TREX-2 and NXF-1 : NXT-1 may together recruit the *hsp-16.2 /41* promoter. However, we note that two of our *nxf-1* alleles ( *yak64* and *yak63* ) are located in N-terminal regions of this protein, while a C-terminal fragment of human NXF1 carrying just its NTF2 and UBA domains was sufficient for binding to GANP (Wickramasinghe et al., 2010) .

The *nxf-1* and *nxt-1* mutants we isolated are overtly wild-type. By contrast, loss-of-function mutations or RNAi knockdown of *nxf-1* and *nxt-1* cause severe defects in mRNA export and lethality (Tan et al., 2000; Zheleva et al., 2019) , suggesting that our mutations may specifically perturb recruitment of the *hsp-16.41* promoter without affecting the general mRNA export functions of NXF-1 : NXT-1 .

## Methods

*Strain maintenance: C. elegans* worms were maintained at 20°C or room temperature (~23°C) on NGM agar plates seeded with lawns of OP50 bacteria (Brenner, 1974) .

*Suppressor screen:* Strains XZ1174 and XZ1372, which both contain two single-copy insertions of *P hsp-16.41 :: peel-1* , were mutagenized with ENU or EMS as described (Brenner, 1974; De Stasio & Dorman, 2001) . F2 animals were heat-shocked at 34° for two hours, and survivors were isolated. *yak63* was isolated from EMS mutagenesis, while *yak64* , *yak65* , and *yak135* were isolated from ENU mutagenesis. All of these mutations are recessive.

*Gene identification:* Mutations were mapped to chromosomes using marker strains EG8040 and EG8041 (Frøkjær-Jensen et al., 2014). *yak63 , yak64 ,* and *yak65* were mapped to chromosome V, and *yak135* was mapped to chromosome I. A complementation test suggested that *yak64* and *yak65* belong to the same gene. *yak63 , yak64 ,* and *yak65* were outcrossed five times, and *yak135* three times using strain XZ1047 to generate strains XZ63, XZ64, XZ65, and XZ135. Genomic DNA was isolated from these strains using the Hobert lab protocol found at http://hobertlab.org/wp-content/uploads/2013/02/Worm_Genomic_DNA_Prep.pdf . Whole-genome sequencing was performed by Novogene and the data were analyzed using the Galaxy web platform (version 18.09) at https://usegalaxy.org/ . Mutations were confirmed by Sanger sequencing. The *nxf-1* mutations are as follows: *yak63* is a C to T mutation leading to P286S, *yak64* is a C to T mutation leading to L101F, and *yak65* is a G to A mutation leading to R486K. The *nxt-1* mutation *yak135* is a T to G mutation leading to I75S.

*Heat-shock:* 50 gravid adults of strains XZ63, XZ64, XZ65, and XZ135 along with controls XZ1174 or XZ1372 were placed on new plates at 34°C for two hours and then moved to 20°C or room temperature. Animals were probed gently by worm pick, and response to touch was measured either as movement or no movement. Animals were assayed immediately following heat-shock and then every thirty minutes for three hours, with a final measurement at 24 hours. This experiment was performed a minimum of three times for each strain.

*Endogenous peel-1 suppression assays:* To test whether *nxf-1 ( yak65 )* suppressed endogenously expressed *peel-1* , we first generated strain XZ2281 that is homozygous for *nxf-1 ( yak65 )* and in which the *zeel-1 ( tm3419 )* deletion is balanced by the hT2 translocation carrying a GFP marker ( *qIs48* ) and a recessive lethal mutation. Similar to the control strain QX1319 *zeel-1 / hT2 [let GFP]* , we found that all XZ2281 surviving animals had GFP, indicating that *zeel-1 / zeel-1 ; nxf-1 ( yak65 )* animals are inviable and that *nxf-1 ( yak65 )* does not suppress endogenous *peel-1* . To better quantify this result, animals of genotype *zeel-1 ( tm3419 )/ oxTi638 [Peft-3::tdTomato::H2B]; nxf-1 ( yak65 )* were constructed via a series of crosses: N2 X EG7832 X EG7766 X XZ65 X XZ2281. Nine *zeel-1 ( tm3419 )/+ ; nxf-1 ( yak65 )* L4 hermaphrodites were grown individually and allowed to produce progeny. The number of dead progeny over the total number of progeny was used to quantify PEEL-1 -induced lethality. For control, AFS216 *zeel-1 ( tm3419 ) peel-1 ( cle6 )* , was crossed to N2 males. AFS216 contains the same *zeel-1 ( tm3419 )* deletion, as well as a *peel-1* early stop-codon insertion and frameshift that is irrelevant for this cross. Seven L4 hermaphrodites produced from this cross were singled to new plates and live and dead progeny were counted.

*P hsp-16.41 ::GFP fluorescence assays:* Strains XZ2418 and XZ2457 carrying a single-copy insertion of *P hsp-16.41 ::eGFP::H2B* were heat-shocked for 2 hours at 34°C. Animals were then placed at room temperature for 2 hours before mounting to agarose slides with 50 mM sodium azide. Entire animals were imaged using a NikonTi2-E Crest X-light V2 spinning disk confocal microscope by excitation at 488 nm and capture with a 510/20 filter. Each image was taken as a Z series with 0.8 μm steps. Images were analyzed using FIJI utilizing the Z-project sum slices tool (Schindelin et al., 2012) . ROIs were drawn around each animal and measured for mean gray value. Background was subtracted from these values using the same ROIs placed outside of the animal. Control worm mean-gray values after background subtraction were averaged for each replicate and used to normalize all values for that replicate.

## Reagents

**Reagents:**

**Table:** 

**Strain**	**Genotype**	**Source**
N2	wild-type *Caenorhabditis elegans*	CGC
XZ63	*oxSi507 [P hsp-16.41 :: peel-1 , Cb-unc-119 ] II ; oxSi280 [P hsp-16.41 :: peel-1 , Cb-unc-119 ] IV ; nxf-1 ( yak63 ) V *	This study
XZ64	*oxSi507 [P hsp-16.41 :: peel-1 , Cb-unc-119 ] II ; oxSi280 [P hsp-16.41 :: peel-1 , Cb-unc-119 ] IV ; nxf-1 ( yak64 ) V *	This study
XZ65	*oxSi507 [P hsp-16.41 :: peel-1 , Cb-unc-119 ] II ; oxSi280 [P hsp-16.41 :: peel-1 , Cb-unc-119 ] IV ; nxf-1 ( yak65 ) V *	This study
XZ135	*nxt-1 ( yak135 ) I ; oxSi507 [P hsp-16.41 :: peel-1 , Cb-unc-119 ] II ; oxSi280 [P hsp-16.41 :: peel-1 , Cb-unc-119 ] IV ; him-5 ( e1490 ) V *	This study
XZ1047	*oxSi507 [P hsp-16.41 :: peel-1 , Cb-unc-119 ] II ; unc-119 ( ed9 ) III ; oxSi280 [P hsp-16.41 :: peel-1 , Cb-unc-119 ] IV ; him-5 ( e1490 ) V *	This study
XZ1174	*oxSi507 [P hsp-16.41 :: peel-1 , Cb-unc-119 ] II ; oxSi280 [P hsp-16.41 :: peel-1 , Cb-unc-119 ] IV *	This study
XZ1372	*yakTi4 [P hsp-16.41 ::eGFP::H2B, NeoR] I ; oxSi507 [P hsp-16.41 :: peel-1 , Cb-unc-119 ] II ; oxSi280 [P hsp-16.41 :: peel-1 , Cb-unc-119 ] IV *	This study
XZ2281	*zeel-1 ( tm3419 ) I / hT2 [ bli-4 ( e937 ) let-?( q782 ) qIs48 ] I; III ; nxf-1 ( yak65 ) V *	This study
XZ2297	*nxf-1 ( yak65 ) V *	This study
XZ2418	*nxf-1 ( yak65 ) V; yakTi6 [P hsp-16.41 ::eGFP::H2B, NeoR] *	This study
XZ2457	*yakTi6 [P hsp-16.41 ::eGFP::H2B, NeoR] *	This study
XZ2463	*unc-119 ( ed9 ) III ; nxf-1 ( yak65 ) V; oxEx1504 [P exp-3 :: peel-1 Cb-unc-119 , Pmyo-3::mCherry, Pmyo-2::mCherry, Prab-3::mCherry] *	This study
EG6301	*qqIr5 [ niDf9 zeel-1 (-) peel-1 (-)]; ttTi5605 II ; unc-119 ( ed9 ) III ; oxEx1504 [P exp-3 :: peel-1 Cb-unc-119 , Pmyo-3::mCherry, Pmyo-2::mCherry, Prab-3::mCherry] *	Erik Jorgensen
EG7766	*oxTi77 [Peft-3::GFP::H2B unc-18 (+)] V ; unc-18 ( md299 ) X* )	Erik Jorgensen
EG7832	*oxTi638 [Peft-3::tdTomato::H2B cb- unc-119 (+)] I ; unc-119 ( ed3 ) III *	CGC
EG7833	*oxTi559 [Peft-3::tdTomato::H2B cb- unc-119 (+)] I ; unc-119 ( ed3 ) III *	CGC
EG7958	*unc-119 ( ed3 ) III; oxTi710 [Peft-3::tdTomato::H2B Cb-unc-119 (+)] V *	CGC
EG8040	*oxTi302 [Peft-3::mCherry cb- unc-119 (+)] I ; oxTi75 [Peft-3::GFP::H2B unc-18 (+)] II ; oxTi411 [Peft-3::tdTomato::H2B cb- unc-119 (+)] III unc-119 ( ed3 ) III; him-8 ( e1489 ) IV *	CGC
EG8041	*oxTi76 [Peft-3::GFP::H2B unc-18 (+)] IV ; oxTi405 [Peft-3::tdTomato::H2B cb- unc-119 (+)] V him-5 ( e1490 ) V ; oxTi421 [Peft-3::mCherry cb- unc-119 (+)] X *	CGC
AFS216	*zeel-1 ( tm3419 ) I peel-1 ( cle6 ) I *	Aaron Severson
QX1319	*zeel-1 ( tm3419 ) I / hT2 [ bli-4 ( e937 ) let-?( q782 ) qIs48 ] I; III *	Leonid Kruglyak
